# Point of View: A Holistic Four-Interface Conceptual Model for Personalizing Shock Resuscitation

**DOI:** 10.3390/jpm15050207

**Published:** 2025-05-20

**Authors:** Philippe Rola, Eduardo Kattan, Matthew T. Siuba, Korbin Haycock, Sara Crager, Rory Spiegel, Max Hockstein, Vimal Bhardwaj, Ashley Miller, Jon-Emile Kenny, Gustavo A. Ospina-Tascón, Glenn Hernandez

**Affiliations:** 1 Intensive Care Unit, Santa Cabrini Hospital, CIUSSS EMTL, University of Montreal, Montreal, QC H1T1P7, Canada; 2 Departamento de Medicina Intensiva, Facultad de Medicina, Pontificia Universidad Católica de Chile, Santiago 8331150, Chile; e.kattan@gmail.com (E.K.); glennguru@gmail.com (G.H.); 3 The Latin American Intensive Care Network (LIVEN); gusospin@gmail.com; 4 Department of Critical Care Medicine, Integrated Hospital Care Institute, Cleveland Clinic, Cleveland, OH 44106, USA; siubam@ccf.org; 5 Departments of Emergency Medicine, Riverside University Health System Medical System, Moreno Valley, CA 92555, USA; khaycockmd@hotmail.com; 6 Loma Linda University Medical Center, Loma Linda, CA 92354, USA; 7 Desert Regional Medical Center, Palm Springs, CA 92262, USA; 8 Departments of Critical Care and Emergency Medicine, University of California Los Angeles, Los Angeles, CA 90095, USA; sara.crager@gmail.com; 9 Departments of Critical Care and Emergency Medicine, Medstar Washington Hospital Center, Washington, DC 20010, USA; rspiegs@gmail.com (R.S.); max.hockstein@gmail.com (M.H.); 10 FNB Critical Care, Narayana Health City, Bangalore 560099, India; vmlbhardwaj@yahoo.co.in; 11 Shrewsbury and Telford Hospitals, Shrewsbury SY3 8XQ, UK; ashleymiller@nhs.net; 12 Health Sciences North Research Institute, Sudbury, ON P3E 5J1, Canada; jon.emile.kenny@gmail.com; 13 Flosonics Medical, Toronto, ON M5V 2Y1, Canada; 14 Department of Intensive Care, Fundación Valle del Lili, Cali 760032, Colombia; 15 Translational Research Laboratory in Critical Care Medicine (TransLab-CCM), Universidad Icesi, Cali 760031, Colombia

**Keywords:** shock, resuscitation, sepsis, microcirculation, hemodynamics, coherence

## Abstract

The resuscitation of a patient in shock is a highly complex endeavor that should go beyond normalizing mean arterial pressure and protocolized fluid loading. We propose a holistic, four-interface conceptual model of shock that we believe can benefit both clinicians at the bedside and researchers. The four circulatory interfaces whose uncoupling results in shock are as follows: the left ventricle to arterial, the arterial to capillary, the capillary to venular, and finally the right ventricle to pulmonary artery. We review the pathophysiology and clinical consequences behind the uncoupling of these interfaces, as well as how to assess them, and propose a strategy for approaching a patient in shock. Bedside assessment of shock may include these critical interfaces in order to avoid hemodynamic incoherence and to focus on microcirculatory restoration rather than simply mean arterial pressure. The purpose of this model is to serve as a mental model for learners as well as a framework for further resuscitation research that incorporates these concepts.

## 1. Background

The diagnosis and management of shock remains challenging [1]. While there has been a strong movement to homogenize care by way of protocols, patients are highly heterogeneous, and many clinicians, including the authors, interpret the current literature as strongly suggestive that a more physiologically personalized approach may benefit outcomes [2].

Over the past two decades, most guidelines have emphasized mean arterial pressure (MAP) targets, with weight-based fluids, followed by vasopressors and inotropes in cases of ongoing hypoperfusion. More recently, attention to fluid responsiveness has led some to use the lack thereof as a fluid stop point, but without assessing potential venous congestion or fluid tolerance [3,4]. While the use of bedside ultrasound has been slowly increasing, it is not yet formally integrated into the majority of resuscitation algorithms or common practice [5].

Recently, alternative resuscitation algorithms have emerged, some focusing on markers of forward flow using echocardiography [6,7], and others assessing peripheral perfusion via capillary refill time (CRT) [8,9]. Nevertheless, the value of a multimodal perfusion assessment has been emphasized by recent guidelines and position papers, including the incorporation of critical variables such as venous–arterial pCO_2_ gradients and central-to-venous oxygen saturation, in addition to CRT, into the decision tree [10,11].

Fortunately, these developments are moving towards a more personalized, goal-oriented approach [12]. The venous side of the circulation has traditionally received very little focus compared to the arterial side. Recently, work surrounding the role of central venous pressure (CVP) in resuscitation [13], as well as ultrasound markers of venous congestion and right ventricular failure, have begun to bring this forward, despite these notions appearing in the literature nearly a century ago [14]. Even the choice of specific vasoactive agents is being studied as a personalization strategy, due to the mechanistic differences between the molecules [15,16].

Hemodynamic coherence refers to the coupling of macro- and microcirculation [17]. Coherence is achieved when manipulation of macro-circulatory variables such as MAP and cardiac output (CO) leads to improvements in microcirculatory flow and tissue perfusion. Conversely, hemodynamic incoherence is the failure of microcirculatory flow despite improved macrohemodynamic parameters. At this stage, excessive fluid or vasoactive administration may in fact worsen tissue perfusion and organ function.

## 2. Conceptual Purpose

In the authors’ experience, advanced resuscitationists perform assessments and re-assessments of each interface in the resuscitation process, but often not in a deliberate fashion, and while they routinely share their knowledge with trainees, it is most often in a piecemeal fashion and not in a holistic, integrated way. The synthesis of this interface-based approach was developed over a series of discussions among the authors to distill what experts believe are the most important considerations in a holistic approach to hemodynamics. Teaching a consistent synthesis would benefit trainees and anyone seeking to learn about hemodynamics. In addition, we hope that such an approach can prompt efforts in research and development of personalized resuscitative strategies.

## 3. Personalizing Resuscitation to Patient Pathophysiology

The authors believe that a focus on MAP or stroke volume (SV), when paired only with surrogate markers of perfusion such as lactate, is insufficient. We propose a four-interface model of the macro- and microcirculation for the assessment and treatment of hemodynamic instability, and for the identification of potential incoherence. For instance, the focus on increasing MAP using crystalloids—while ignoring that the increase in central venous pressure can itself decrease tissue perfusion pressure—may worsen patient outcomes, as further discussed below [18]. Similarly, increasing MAP with vasopressors without realizing that the increased afterload may decrease SV in a failing heart [19], and thus tissue perfusion—may be equally deleterious, albeit from a different mechanism. Hence, clinicians should familiarize themselves and be able to assess each of these key hemodynamic interfaces.

For the purposes of this conceptualization, source control is both paramount and assumed. No amount of personalized resuscitative efforts will improve patient outcomes if source control is not achieved in parallel. As such, prior to engaging in any phenotyping of a patient’s shock, it is necessary to have excluded cardio-respiratory mechanical causes of shock, which include pathologies such as massive pulmonary embolism, ventricular free wall rupture, papillary muscle rupture, tamponade, dynamic left or right ventricular outflow tract obstructions, acute ventricular septal defect, tension pneumothorax, acute myocardial infarction, severe air trapping, abdominal compartment syndrome, and other pathologies that have specific treatments.

This hemodynamic conceptualization therefore applies to the optimization of the patient in shock without any interventionally reversible cause, or as a bridge until true source control is achieved. We propose the following four critical shock-related interfaces within the circulatory system: (I) left ventricle (LV) to systemic arterial, (II) arteriolar to capillary (macro- to microcirculation), (III) capillary to venular, and (IV) right ventricle to pulmonary artery (RV to PA).

**Figure 1:**
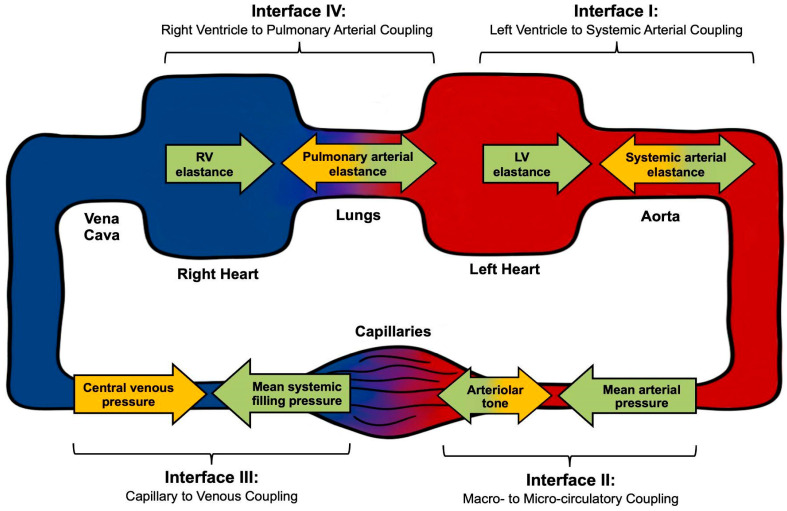
Diagrammatic representation of the four main circulatory interfaces.

Note that there are other important hemodynamic interfaces—the veins to large “terminal” veins (inferior and superior venae cavae), the large veins to the right atrium, as well as the pulmonary venous system to the left atrium. While such additional variables are not included in the core conceptual model outlined here to avoid making it overly cumbersome, the four proposed interfaces can be easily assimilated into a broader hemodynamic framework, as outlined in Figure 1.

We offer an integrated framework for assessing the above-described interfaces at different levels, ranging from a simple, minimal-resource approach to a full-technology one, serving as a mental model for both clinical use and research design.

## 4. Circulatory Coupling

“Coupling” refers to the unique structures between which energy transfer occurs. Coupling is considered ideal when it results in minimal expenditure and maximal efficiency. Hemodynamic coupling, historically, has been quantified as a ratio of elastances (change in pressure per change in volume): arterial tree elastance (Ea) divided by the elastance of the ventricle (Ees) [20,21]. Arterial elastance is a determinant of LV afterload, whereas ventricular elastance is a marker of contractility. Thus, Ea/Ees is a quotient demonstrating the balance between afterload and contractility. Take, for example, a patient presenting in vasodilatory shock from sepsis. Upon initial assessment, the left ventricular ejection fraction (LVEF) appears to be normal, but Ea, or afterload, is low due to vasodilation. A vasopressor is started to counter the low resistance and improve the left ventriculo–arterial coupling. On reassessment, once normal arterial resistance has been restored, impaired LV function is noted, and a decreased contractility that was initially masked by the low afterload. To re-establish left ventriculo–arterial coupling, an inotrope may be required to improve contractility and therefore LVEF.

Coupling can be graphically depicted on a Cartesian plane, plotting volume on the abscissa and pressure on the ordinate [20]. The resulting line, the end-systolic pressure–volume relationship (ESPVR), is a marker of the LV inotropic state. The ESPVR line’s intercept on the ordinate is termed V_0_, a theoretical state where the left ventricle is completely decompressed. Significant deviation from coupling indices, or uncoupling, result in clinically evident circulatory pathology. In this model, we include circulatory interfaces that do not include a pump per se, but where the pressure differences nonetheless possess the potential for uncoupling.

## 5. The Concept of Mean Systemic Filling Pressure (MSFP)

During circulatory flow, pressures in the arterial and venous circulation are dissimilar due to differences in the compliance of the arterial and venous compartments. If circulatory flow is stopped, pressure rapidly equalizes between the two compartments as blood volume moves from the arterial compartment to the venous side down a pressure gradient—until the gradient is exterminated. The equalization systemic pressure at “stop flow” conditions is the MSFP, and is normally about 7–10 mmHg in mammals [22,23,24]. The factors influencing MSFP include the stressed volume (volume that distends the vasculature) and the sum total of venous compliance or venous tone. Thus, the MSFP will increase or decrease with changes in either intravascular volume or vascular tone. MSFP is the driver behind venous return (VR), with the CVP being the impeding downstream pressure to flow [24]. It is important to note that MSFP is not only a preloading force for the cardiovascular system but also an afterload to organ venous flow.

## 6. The Relevance of Microhemodynamic Variables

Intuitively, the difference between MAP and CVP should govern blood flow in the major cardiovascular circuit and has commonly been referred to as perfusion pressure. However, this assumption would be true if the vascular system were a continuous and rigid tube, which it is not [25]. Indeed, the hydraulic transition from macro- to microcirculation involves a series of intricate phenomena. Microvascular flow remains nearly constant across a wide range of pressures, as tissues can adapt their own perfusion to match oxygen and cell metabolic demands. Such local regulation of flow is finally governed by a combination of cellular and endothelial signals (including oxygen, potassium, hydrogen ions, lactate, adenosine, inorganic phosphate, prostanoids, eicosanoids, endothelium-derived nitric oxide, among others), as well as sympathetic influx and vascular myogenic responses [26], which will not be discussed in this manuscript.

The contractile action of the heart provides the force for the bloodstream, which is transmitted along the vascular tree as a pressure wave and then gradually dissipated by the resistance encountered as vessels subdivide and narrow. This leads to a rapid drop in arterial pressure within the small arterioles as a function of resistance. Interestingly, the steepest drop of pressure in the systemic circulation occurs in the arterioles, whose aggregated resistance is higher than the sum of capillary resistances.

According to Laplace’s Law, vascular tension depends on the balance between the distending force generated by the transmural pressure that pushes the wall outward, and the constricting force from elastic components within the wall that pulls it inward. When vasomotor tone or external forces exceed local arterial pressure (i.e., when transmural pressure becomes negative), the vessel collapses, thus limiting flow. The intraluminal pressure at which arterial vessels collapse is the so-called critical closing pressure (Pcc), which would represent the effective back pressure to arterial flow. In other words, the difference between MAP and Pcc should represent the tissue perfusion pressure (TPP).

Several observations have suggested a “non-continuity” of the vascular system, reflected by significant discrepancies between pressures registered during flow cessation at the arterial and venous side of the circulation, in both animal models [27,28] and humans after spontaneous cardiac arrest [29]. Such arterial-to-venous pressure gradient at zero flow represents the so-called vascular waterfall (VW) [30], which theoretically functions to keep arterial pressure slightly elevated, potentially sustaining blood flow to vital organs [31,32]. Such VW could indeed be explained by a Starling resistor-like mechanism [31]. Some authors have theorized that, just as in a waterfall in which flow over the edge is theoretically independent of the height of the fall itself, flow beyond the Pcc point should be independent of outflow pressure, i.e., independent of further downstream capillary and venous pressures. Nevertheless, this last concept has not been clearly proven in both humans and even in vivo models.

Progress in bringing these physiological concepts to the clinical arena has been hindered by the challenge of bedside measurement. Beneficial or detrimental effects of vasopressors on tissue perfusion can occur depending on their relative actions on MAP and CCP [33]. The ability to monitor TPP would offer an advantage for MAP optimization in circulatory shock patients [34].

In the discussion below, we will refer to several monitoring and assessment techniques, which we will not expand on in detail, as an in-depth review and validation of these is beyond the scope of this text. Interested clinicians should familiarize themselves with the techniques, application, and limitations of each of these before application.

### 6.1. Interface I: Left Ventriculo-Arterial Coupling—End-Systolic Elastance and Systemic Arterial Elastance

Coupling at this interface is expressed as the ratio of effective Ea to the end-systolic elastance of the LV, or Ea/Ees. The gold standard for assessment of ventriculo–arterial coupling (VAC) requires invasive catheterization of the left ventricle and simultaneously measuring ventricular volume (by conductance) and pressure (by pressure transduction) measurements to create the slope of the ESPVR [35,36]. The invasive nature along with the complexity of the multi-beat process is too cumbersome for routine use. Therefore, there have been alternative approaches devised, using a single-beat method, to estimate Ea/Ees. Chen et al. devised one for estimating Ees using systolic and diastolic blood pressure measurements, combined with echocardiography to obtain stroke volume, EF, pre-ejection time, and total ejection time [37]. Ea can be estimated by dividing 90% of the systolic blood pressure (SBP) by stroke volume (SV) using the following formula: Ea = (0.9 × SBP)/SV. The Chen algorithm has been even integrated into a free app (iElastance) to facilitate its calculation. In addition, Monge Garcia et al. showed in an experimental study that changes in Eadyn (pulse pressure variation/stroke volume variation) reflect changes in VAC [38].

There are limitations to single-beat estimation. When V_0_ is zero, Ea/Ees can more simply be described in terms of the LVEF [39]. Anyone measuring LVEF should know it reflects VAC; LVEF is not a measure of contractility, as it depends on loading conditions and end-diastolic volume. It becomes more useful in shock management when considered in the context of Ea. For example, if LVEF (and therefore VAC) is normal when Ea is low in a patient with vasodilatory shock, increasing Ea with a vasopressor may result in a decrease in LVEF, revealing underlying impaired LV function—an inotrope may then be required. A high LVEF and low Ea is a sign of a hyperdynamic circulation, usually seen in resuscitated vasodilatory shock [20,40].

It is important to note that hypovolemia represents a form of interface I failure due to low LV preload and SV, even if there is no technical “uncoupling”, as Ees and Ea are both increased with sympathetic activation, so the Ea/Ees ratio may remain normal. Ultimately, proper function at interface I requires coupling as well as adequate SV (hence adequate preload) and CO. It is particularly relevant to consider that the term “adequate” SV or CO must be determined by its effectiveness to achieve or maintain normal tissue perfusion.

Once an uncoupled state is identified, the clinician must then decide how to address the Ea and/or the Ees and SV. If a low preload is detected, causing a low SV, this needs to be corrected, which may require fluids if the patient is frankly hypovolemic, vasopressors to restore MAP, DBP, and eventually MSFP if there is a vasodilatory issue, or assistance to the RV if LV preload is in fact impaired by RV dilation/limitation/septal shift. Additionally, reassessment of VAC after any therapeutic intervention is prudent and necessary.

#### Interface I at the Bedside

Point of care ultrasound (POCUS) enables the clinician to obtain LVEF, which is the most closely related echographic parameter, as it is inherently a load-dependent variable—hence, an inherent measure of coupling between contractility (Ees) and afterload (Ea). Thus, a normal LVEF essentially rules out uncoupling. However, it does not necessarily equate to an adequate CO (which requires an adequate SV, not only a normal EF). Left ventricular outflow tract velocity time integral (LVOT-VTI) correlates (and can be used to measure) SV, such that an adequate VTI (>18 cm^2^) likely rules out significant uncoupling.

In the absence of POCUS availability, pulse pressure (PP) can provide a correlation to SV, although it is subject to false positive error in patients with increased afterload or low aortic compliance due to reflected pressure waves. In addition, a low PP may reflect either profound hypovolemia or LV failure. Corrected flow time of the carotid artery (cCFT) is also proposed as an SV surrogate because the duration of mechanical systole is directly proportional to SV [41,42]. While a cCFT greater than approximately 300 milliseconds (ms) is normal [43], this threshold could lead false positive results when Ea is elevated and/or Ees is diminished, both of which prolong ejection for a given SV; like PP, the duration of ejection increases with age [44,45].

### 6.2. Interface II: Arterioles to Capillary

**Figure 2:**
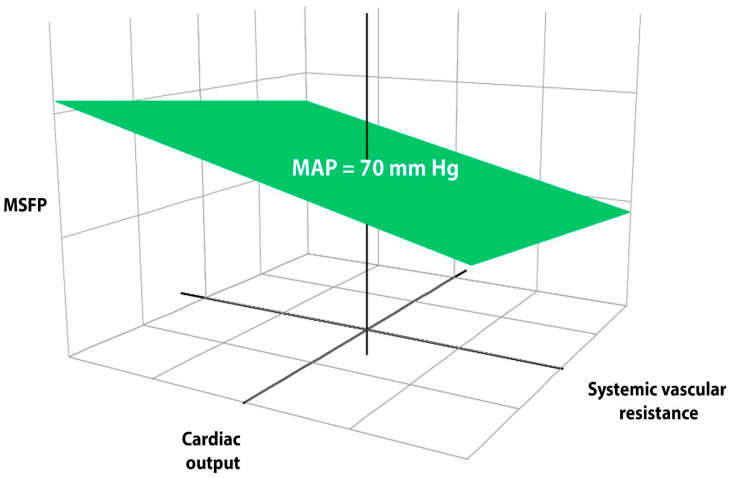
Theoretical response surface model for a given mean arterial pressure (70 mmHg) according to different combinations of vascular resistance, cardiac output, and preload. MAP: mean arterial pressure; MSFP: mean systemic filling pressure. Note that cardiac output can be adequate or inadequate for the same MAP when it is maintained by a higher resistance.

The second interface occurs at the distal part of the arterial system where Pcc occurs, interfacing with the capillary network [46]. Uncoupling can occur if excessive vasoconstriction limits capillary perfusion, which can happen when MAP is low (due to low SV), but may also happen in the presence of a normal or even elevated MAP driven significantly by vasoconstriction with a concomitant elevation of the Pcc (Figure 2 with high arterial resistance). The relevance of the DBP is often overlooked. With increased compliance of the proximal large arteries and increased distal resistor tone of the arterioles, the DBP is increased [47]. Faster heart rates also promote higher DBP, as there is less time for the pressure to decay before a reloading volume—and pressure—is delivered by the next systolic period [48]. As about ⅔ of the time of a cardiac cycle is spent in diastole, it is important that DBP is maintained above the Pcc of the major capillary beds; otherwise, the viscoelastic properties of the capillary beds will cause them to collapse, resulting in uncoupling of interface II. Normally, local control of regional arteriolar tone allows tissue beds to recruit more or less of the MAP to satisfy their various metabolic needs. However, this local control is often compromised in shock states—either by local pathological derangements or by inappropriate prescription of various therapies, such as excessive vasoconstriction.

It is important to understand that the relationship between MAP and tissue perfusion is non-linear. In Figure 2, one can visualize that a patient can be on the iso-pressure plane but, if in the right lower quadrant, CO is low and arterial resistance high. Hence, the critical closing pressure will also be high, and perfusion will have dropped off despite a normal MAP. Not all MAPs are created equal.

The emergence and validation of CRT as a resuscitation target—supported by strong epidemiological data [49], physiological background [50], and a major RCT—has led to its use as a surrogate of microcirculatory perfusion [9,51]. With its extremely rapid response to potentially flow-increasing maneuvers (fluid or MAP challenges), it is the ideal variable to assess the status of macro-to microcirculatory coupling [8,52,53]. In addition, it is simple, rapid, extremely low-cost, and requires no technology. However, like any perfusion monitoring variable, it has some drawbacks, including interrater reliability, gaps in physiological background knowledge, and the impact on CRT assessment of high or changing vasopressor doses, etc.

Another potential measure of tissue perfusion that is equally simple and available is the skin mottling score, which has been strongly associated with mortality in sepsis and cardiogenic shock [54,55,56].

To some degree, tissue perfusion can also be coarsely assessed by global markers, such as the deltas between arterial and venous (central/mixed) and tissue saturation (StO_2_), representing an “adequacy of supply” measure. A pCO_2_ gap at or below 6 is generally considered suggestive of adequate CO for demands [57]. In addition, analysis of the recovery slope after a vascular occlusion test with thenar NIRS may disclose the status of microvascular reactivity.

#### Interface II at the Bedside

It is very complex to evaluate this interface at the bedside since only surrogates of microcirculatory flow can be used. Obviously, when real-time assessment of organ function is possible (e.g., brain and mental status), this is ideal; however, this is not always possible in critically ill patients. Extensive research during the last two decades using handheld videomicroscopes in the sublingual area has described several abnormalities in flow, density, and heterogeneity at this level, some of which some are related to shock-related endothelial dysfunction. However, this technique is expensive and restricted to the research arena, though it may soon become available to clinicians.

As of now, and as established by the ANDROMEDA study, capillary refill time is the only evidence-based practical tissue perfusion surrogate [9]. Some recent studies suggest that the response of CRT—representing an extensive microcirculatory territory—to a fluid or a MAP challenge may disclose the status of hemodynamic coherence. A decrease of CRT of more than 25% or one second immediately after a fluid bolus, or an increase in MAP to 80–85 mmHg for 30 min, may signal a preserved coherence, meaning that improvement in the macrocirculatory variables will improve microcirculatory perfusion [8,52,53].

### 6.3. Interface III: Distal Capillary to Venular

At this level, vascular pressures are low, operating near MSFP values. The gradient driving flow from the tissues after the VW is the gradient between MSFP and CVP; hence, the main factor affecting tissue perfusion post-waterfall is the CVP. This venous side of the circulation has long been overlooked, partly since venous pressures—often an order of magnitude lower than arterial pressures—have been ignored in favor of “forward flow-centrism” and an overly simplified conceptual model of perfusion pressure.

When CVP and, subsequently, venous and venular pressures rise, microcirculatory dysfunction may be induced either by stasis or by a decrease in capillary density secondary to tissue edema [18]. As venular pressures increase, this will inevitably cause stasis and edema, worsening the true perfusion pressure. Though this is physiologically a logical construct, it is important to acknowledge that clinical evidence showing improved outcomes with congestion correction is still under investigation. It has not yet been demonstrated, for example, whether increasing Pmsf by increasing CVP independent of decreasing CO has a specific detrimental effect, or whether removing fluid by diuresis or dialysis restores capillary perfusion and improves organ function and prognosis. This opens new avenues for specific research in this therapeutic area.

The importance of CVP is underscored by studies showing that a high CVP (>12 mmHg) is associated with worse tissue perfusion, as measured by a low microvascular flow index (MFI < 2.6) [18]. More recently, Beaubien-Souligny et al. demonstrated that congestive abnormalities in solid organ venous Doppler correlated with organ dysfunction in post-op cardiac surgery patients [58]. This has since been replicated in several studies, establishing the Venous Excess Ultrasound (VExUS) score as a tool to measure the severity of congestion [59,60,61]. This tool must be considered as a starting point and should be validated by ongoing studies in other scenarios, such as septic shock.

It is important to realize that Interface III uncoupling takes place at the organ or tissue level, such that the cause of CVP elevation is immaterial. The presence of a significantly elevated CVP (likely values over 10–12) may uncouple interface III and potentially contribute to tissue hypoperfusion irrespective of the cause. This is the key point of interface III, which reflects the perspective of the tissue beds—venous afterload—as opposed to strictly a circulatory parameter. This is important, as any resuscitative strategy that causes interface III uncoupling can worsen tissue perfusion and organ function, irrespective of potential improvement in macro-hemodynamics—another mechanism behind hemodynamic incoherence. It is probably more appropriate to view CVP as tissue afterload rather than cardiac preload.

#### Interface III at the Bedside

Elevated jugular venous pressure reflects increased CVP. This can be measured by clinical exam or by POCUS [62]. Locating the jugular venous pulse (JVP) allows estimation of the central venous pressure (CVP) and inference of right heart hemodynamics by analyzing the x’ and y descents, as well as the a and v waves. When supine, a larger jugular Doppler systolic (S) wave than diastolic (D) wave is normal, recapitulating x’ > y descent. Several studies have shown that S = D, S < D, and monophasic D wave filling are abnormal patterns associated with RV dysfunction, tricuspid regurgitation, and/or pulmonary hypertension [63,64,65,66].

Femoral vein Doppler (FVD) is another tool to assess the effect of central venous pressure elevation [67]. It is considered suggestive of venous congestion if any of the following criteria are fulfilled: (1) pulsatile in nature, (2) retrograde flow velocity of more than 10 cm/s, or (3) flow reversal/retrograde flow velocity being more than 1/3rd of antegrade flow velocity. It demonstrates a moderate level of agreement and high sensitivity in detecting elevated CVP levels (>12 mmHg). Doppler envelope of abdominal organs has also shown a close correlation with central venous pressures and, more importantly, with organ dysfunction. Both the VExUS score—a composite of IVC, hepatic, portal, and intra-renal venous assessment—and the renal venous stasis index (RVSI) are associated with organ dysfunction as congestion increases [68].

Further studies should address whether the risk of acute kidney injury (AKI) is better predicted by a high CVP or by the mean perfusion pressure (MAP-CVP), as compared with the VexUS score, considering a recent negative study with the latter [59,69,70,71].

### 6.4. Interface IV—Right Ventricular (RV) to Pulmonary Arterial (RV-PA)

Excessive fluid administration and/or uncoupling of the RV-PA interface is what leads to an elevated CVP (aside from mechanical issues affecting the right atrium, such as tamponade, tension pneumothorax, etc.). If the uncoupling is severe enough to elevate CVP, it may uncouple interface III, emphasizing the tight linkage between these interfaces. However, while the assessment of interface III focuses on the effects on the tissues, interface IV assessment is intended to diagnose and guide treatment of the cause of RV-PA uncoupling.

RV-PA coupling is defined as Ees/Ea, rather than Ea/Ees, and differs from LV–arterial coupling in several ways. The normal RV has a lower Ees compared to the LV and ejects blood into the pulmonary circulation with a lower Ea. Optimal RV-PA coupling occurs at a ratio of about 1.5–2:1. Outside of primary RV cardiomyopathies and RV infarct, RV-PA uncoupling typically results from increased Ea. This is true in chronic cases of pulmonary arterial hypertension, though there is ample time for a compensatory increase in Ees. In acute pulmonary hypertension, the unconditioned RV may not be able to adapt to abrupt rises in Ea. In contrast to metrics such as pulmonary vascular resistance, Ea incorporates both pulsatile and non-pulsatile measures of pulmonary afterload. As such, it can provide information on loading conditions related to left heart function, thereby integrating interfaces I and IV [72,73].

As in LV–arterial coupling, the gold standard method of assessment is performed using invasive conductance catheterization to measure both Ea (calculated as end-systolic pressure divided by stroke volume) and Ees (the slope of the ESPVR curve). Using a standard pulmonary artery catheter (PAC), Ea can be approximated using calculations such as mPAP/stroke volume, but no easily measurable surrogate for Ees exists. The maximal slope of the RV pressure waveform upstroke (dP/dt max) could be considered a rough surrogate for contractility but is inherently load-dependent. Single-beat estimation of Ees, Ea, and the ratio can be performed using the RV pressure tracing as well, but both methods require offline processing of waveform data and are hence impractical for the bedside physician. Non-invasive echographic parameters are used to assess RV-PA coupling, and while they only have a moderate to strong correlation with invasive gold standards, they correlate fairly well prognostically (Siuba, personal communication).

#### Interface IV at the Bedside

Direct measurement of CVP is similar to JVP and, if elevated beyond 10–12 mmHg, supports at least the presence of some degree of uncoupling. Additionally, closer analysis of the CVP waveform can suggest poor RV contractility, diastolic dysfunction, and tricuspid regurgitation [74].

The best available bedside techniques for practical assessment of RV-PA coupling rely on echocardiographic surrogates. The best-validated echocardiographic method for the assessment of RV–PA coupling is the TAPSE/PASP ratio [75]. A TAPSE/PASP ratio of less than 0.31 is specific for RV-PA uncoupling by invasive methods < 0.8 (normal Ees/Ea > 1.5), though cutoffs vary considerably. TAPSE is a simple and relatively accurate estimate of RV EF (EF being related to Ees) and similarly correlates with invasive Ees/Ea. Like EF, it is an inherent reflection of coupling as it is both load- and contractility-dependant. PASP, although influenced by SV and HR, contains similar information as Ea. In fact, a recent study showed, not surprisingly, that an abnormal TAPSE/PASP was a negative prognostic factor for patients in septic shock [72].

Other estimates of RV EF include S’ and FAC. RV S’ employs tissue Doppler techniques to determine tricuspid annular systolic velocities and is a less angle-dependent measure of EF than TAPSE.

Further assessment of RV-PA coupling involves interpretation of RVOT Doppler morphology, including end-diastolic pulmonic regurgitation velocity (PRedv), acceleration time (AT), presence of RVOT VTI notching, and relative pre- and post-notch velocities, if present.

## 7. Integrating the Circuit

After a thorough assessment, the clinician remains faced with the challenge of identifying the weak(est) link(s) among the interfaces to focus treatment and reassess the response at all levels. There is likely to be some degree of trial and error, as it is often difficult to reliably predict the degree of response to therapy, and hence, the critical importance of close monitoring and re-assessment, while remembering that ultimately, tissue perfusion is what matters most.

## 8. Concepts of Clinical Management and Using the Forrester–Kenny Diagram

**Figure 3:**
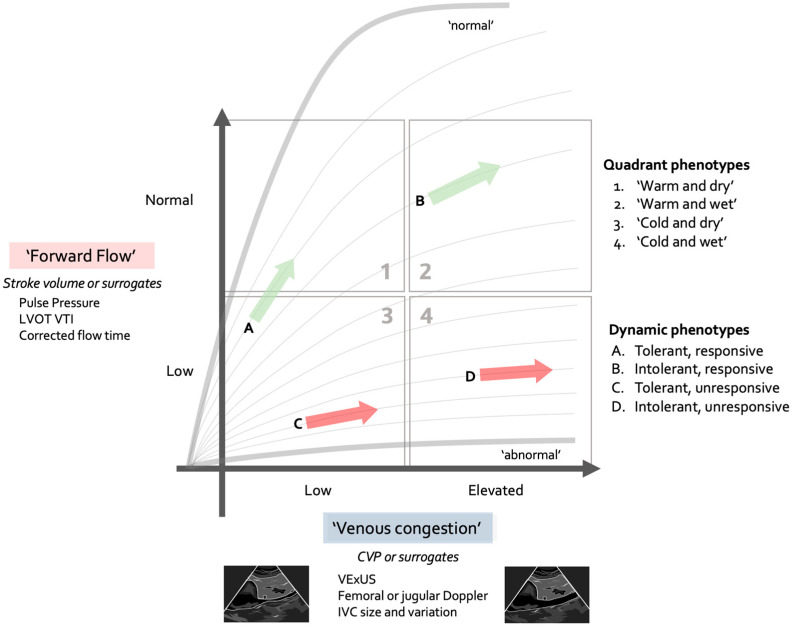
Forrester–Kenny diagram showing the four phenotypic quadrants as well as dynamic phenotypes. Note that different parameters of forward flow and venous congestion may be used depending on available technology and physician familiarity.

Decades ago, Forrester designed a diagram plotting cardiac index against wedge pressure to describe clinical phenotypes post-myocardial infarction [76]. Recently, Kenny reworked the concept, utilizing LVOT VTI on one axis and VEXUS on the other [77]. We believe this is a useful concept that can be used for the very initial assessment of shock patients, and subsequently to track their progress during resuscitation. It can be applied using varying parameters of forward flow vs. congestion, as shown in Figure 3. It is important to acknowledge that this diagram focuses on forward flow and venous congestion, which are fundamental macrohemodynamic variables essential for tailoring resuscitation to restore tissue perfusion in shock states. By intention, and to facilitate clinical applicability, it does not take into account more complex physiological variables such as RV or LV VAC, unless they cause low CO or elevated CVP. Indeed, profound RV or LV failure may occur with compensated and preserved flow and normal CVP, at least at the beginning. However, in practical terms, this diagram can be applied in many clinical situations during the very initial moments of resuscitation, prior to fully assessing the interfaces.

### 8.1. Step 1: Placing the Patient in the Forrester–Kenny Diagram

In the first minutes of the initial assessment, there should be an attempt to place the shock patient into one of the four quadrants, using some measure of congestive assessment on the Y-axis and some measure of forward flow or stroke volume on the X-axis. This should provide the clinician with a preliminary therapeutic strategy. This is conceptually important because patients who are cold generally have a low CO, while those who are warm, even if hypotensive, often have normal or elevated cardiac output. On the other side, patients who are “dry,” meaning without significant evidence of pulmonary congestion or elevated jugular venous pressure, are more likely to be fluid tolerant and responsive, while those who have signs of congestion are less so. While this does not have perfect sensitivity and specificity, it can nonetheless guide the initial therapeutic decision while further assessment is ongoing.

For instance, a patient who would fall in quadrant 2 (warm and wet) should probably receive minimal fluids (which could be harmful by uncoupling interfaces III or IV) and an emphasis instead on vasoconstriction. Conversely, a patient in quadrant 3 (cold and dry) would probably benefit more from avoiding vasoconstriction, which could uncouple interface II, and likely would need fluids and/or inotropes.

### 8.2. Step 2: Assessing the Interfaces

**Figure 4:**
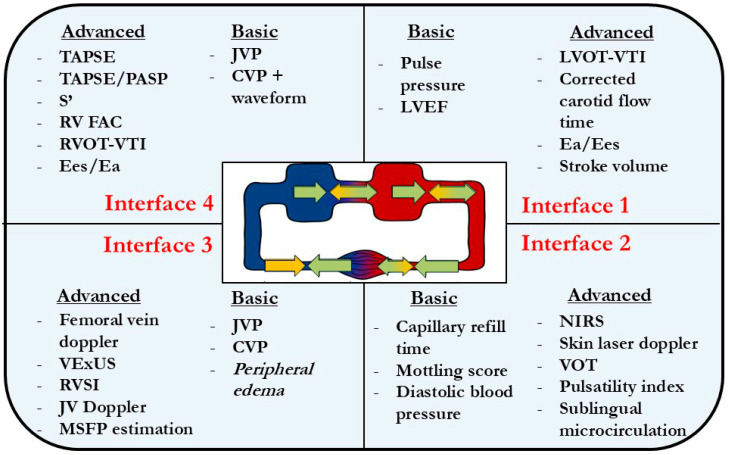
Basic and advanced assessment alternatives for each interface (note that this may evolve with further research to include new parameters). Abbreviations: TAPSE—tricuspid annular plane systolic excursion; PASP—pulmonary artery systolic pressure; S’—tissue Doppler velocity; RV—right ventricle; FAC—fractional area change; Ees/Ea—end-systolic elastance/arterial elastance; JVP—jugular venous pulse; CVP—central venous pressure; LVEF—left ventricular ejection fraction; VTI—velocity time integral; VExUS—venous excess ultrasound; RVSI—renal venous stasis index; JV—jugular venous; MSFP—mean systolic filling pressure; NIRS—near infrared spectroscopy; VOT—vascular occlusion test.

After assessing each interface, the clinician will have to identify the more severely affected one(s) and initiate a therapeutic plan. While closely correlated, uncoupling in interfaces I and IV may not necessarily result in clinically significant uncoupling at the more important interfaces from a perfusion standpoint. For instance, a patient may have a poor LVEF, but maintain a reasonable SV via LV dilation, and have a well-coupled macro- to microcirculation. On the venous side, one may have a very compromised interface IV (e.g., TAPSE/PASP ratio below 0.3 and an elevated JVP), but concomitantly have a VExUS showing mild congestion and only a mildly pulsatile FVD; hence, it is not really uncoupled at interface III. Figure 4 illustrates various means by which the four interfaces can be assessed.

### 8.3. Step 3: Tracking the Progress of Resuscitation on the Forrester–Kenny Diagram

**Figure 5:**
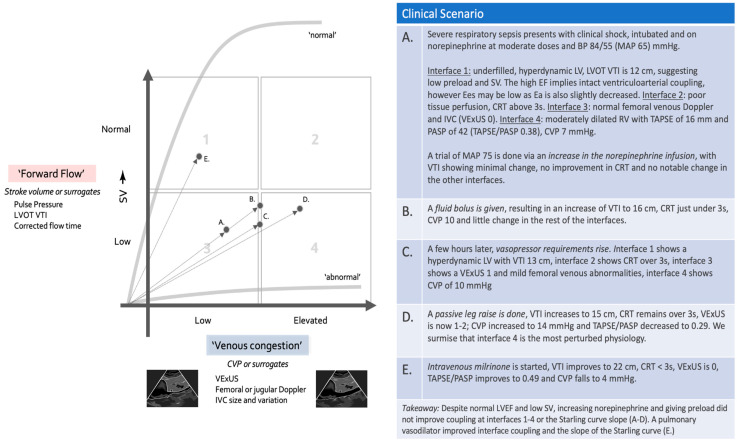
Clinical scenario illustrating the evolution of a patient undergoing resuscitation in a framework of both congestive and forward flow parameters.

Assessing therapy is a key component of a good resuscitation strategy. The patient can be re-plotted on the four-quadrant graph to ensure that he/she is headed towards quadrant 1—warm and dry—where perfusion occurs without congestion and coupling of all interfaces is reasonable. Understandably, this may not be possible in many cases, and the clinician may have to be satisfied with a non-worsening of the clinical path while hoping that, over time, source control and/or tissue healing will remedy the situation, but it is important to ensure that therapeutic interventions are at least not worsening the situation (Figure 5).

## 9. Further Development

There is no question that the relationship between the macro- and microcirculatory systems—interface II—is vital to understanding and managing shock at the bedside. Unfortunately, it is also the interface with the most limited tools to quantify reliably with any granularity. There is a substantial amount of very interesting research that has been conducted, and certainly much is happening in this field, but to date, there is a dearth of tools to evaluate uncoupling that occurs at this level. Furthermore, while the physiological and clinical evidence, which does exist, supports many aspects of a personalized resuscitative strategy, it is important to note that, as a whole, there is no evidence that such an approach improves survival, and evidence-based medicine purists may protest. However, one could argue the same is true for any resuscitative strategy currently employed. In fact, study after study on heterogeneous critically ill patients assessing any particular intervention for shock consistently fails to provide positive results. Such continued quests to find a one-size-fits-all approach to shock betray the complexity of the patients we treat, as well as their underlying acute and chronic physiology. Careful assessment and re-assessment of the four key interfaces is required in most cases, with the ultimate goal of restoring microcirculatory flow and tissue perfusion. We would encourage researchers to go beyond MAP, lactate, and weight-based fluid loading. We hope future research will incorporate a more personalized approach to the management of shock, utilizing the interface principles to seek “perfusion without congestion” in their trial designs.

## 10. Conclusions

A holistic and personalized approach to resuscitation is important in critically ill patients. The authors would also like to remind clinicians that guidelines remain guidelines, and are not, in a rapidly developing field, gold standards. It is paramount to phenotype a patient’s shock, identify its source, and characterize the perturbations in each of the four interfaces described above to avoid unhelpful and even harmful resuscitative measures. While large RCT data with meaningful clinical outcomes is lacking, we feel a four-interface model of shock assessment may represent an adequate compromise of clinical evidence, physiologic reasoning, and clinical efficiency to allow clinicians to appropriately manage the heterogeneity and complexity of these critically ill patients, and an ideal tool for learners to develop a mental model of shock and its management.

## Data Availability

Not applicable.

## Abbreviations

MAP: mean arterial pressure; CVP: central venous pressure; CRT: capillary refill time; CO: cardiac output; SV: stroke volume; LV: left ventricular; RV: right ventricular; PA: pulmonary artery; Ea: arterial elastance: Ees: ventricular elastance; LVEF: left ventricular ejection fraction; SVR: systemic venous resistance; ESPVR: end-systolic pressure–volume relationship; MSFP: mean systemic filling pressure; VR: venous return; Pcc: critical closing pressure; VW: vascular waterfall; TPP: tissue perfusion pressure; VAC: ventriculoarterial coupling; SBP: systolic blood pressure; PP: pulse pressure; POCUS: point of care ultrasound; LVOT-VTI: left ventricular outflow tract velocity time integral; cCFT: corrected flow time of the carotid artery; ms: millisecond; DBP: diastolic blood pressure; CRT: capillary refill time; NIRS: near infrared spectroscopy; STO_2_: tissue saturation; VExUS: Venous Excess Ultrasound; FVD: Femoral vein Doppler; PAC: pulmonary artery catheter; PRedv: end diastolic pulmonic regurgitation velocity; AT: acceleration time; RCT: randomized clinical trial.
